# Risk of Type 2 Diabetes among Osteoarthritis Patients in a Prospective Longitudinal Study

**DOI:** 10.1155/2014/620920

**Published:** 2014-11-04

**Authors:** M. Mushfiqur Rahman, Jolanda Cibere, Aslam H. Anis, Charlie H. Goldsmith, Jacek A. Kopec

**Affiliations:** ^1^School of Population and Public Health, University of British Columbia, Vancouver, BC, Canada V6T 1Z3; ^2^Department of Applied Statistics, East West University, Aftabnagar, Dhaka 1212, Bangladesh; ^3^Department of Medicine, University of British Columbia, Vancouver, BC, Canada V5Z 1M9; ^4^Arthritis Research Centre of Canada, 5591 No. 3 Road, Richmond, BC, Canada V6X 2C7; ^5^Centre for Health Evaluation and Outcome Sciences, Vancouver, BC, Canada V6Z 1Y6; ^6^Health Sciences, Simon Fraser University, Burnaby, BC, Canada V5A 1S6

## Abstract

*Objectives*. Our aim was to determine the risk of diabetes among osteoarthritis (OA) cases in a prospective longitudinal study. *Methods*. Administrative health records of 577,601 randomly selected individuals from British Columbia, Canada, from 1991 to 2009, were analyzed. OA and diabetes cases were identified by checking physician's visits and hospital records. From 1991 to 1996 we documented 19,143 existing OA cases and selected one non-OA individual matched by age, sex, and year of administrative records. Poisson regression and Cox proportional hazards models were fitted to estimate the effects after adjusting for available sociodemographic and medical factors. *Results*. At baseline, the mean age of OA cases was 61 years and 60.5% were women. Over 12 years of mean follow-up, the incidence rate (95% CI) of diabetes was 11.2 (10.90–11.50) per 1000 person years. Adjusted RRs (95% CI) for diabetes were 1.27 (1.15–1.41), 1.21 (1.08–1.35), 1.16 (1.04–1.28), and 0.99 (0.86–1.14) for younger women (age 20–64 years), older women (age ≥ 65 years), younger men, and older men, respectively. *Conclusion*. Younger adults and older women with OA have increased risks of developing diabetes compared to their age-sex matched non-OA counterparts. Further studies are needed to confirm these results and to elucidate the potential mechanisms.

## 1. Introduction

Diabetes mellitus is a common chronic health condition worldwide. It is predicted that the global prevalence of this disease among adults will rise from 6.4% in 2010 to 7.7% by 2030 [1]. Diabetes affects an estimated 8.3% of Americans and 8.8% of Canadians [2, 3], resulting in severe damage to the cardiovascular system, kidneys, eyes, and other organs. Metabolic syndrome is a group of conditions such as hypertension, hyperlipidemia, obesity, and elevated blood glucose that are linked with diabetes [4]. Other common risk factors for diabetes include age, sex, family history, ethnicity, socioeconomic status (SES), heart disease, history of gestational diabetes, physical inactivity, alcohol consumption, and diet [5, 6]. As the prevalence of diabetes has risen, it has been imperative to identify determinants beyond these traditional risk factors. Studies have shown that the increased risk of diabetes is caused in part by physical inactivity and that physically active individuals have lower rates of the disease [6, 7]. In addition, muscle strength was found to be significantly lower among adults with type 2 diabetes [8]. Osteoarthritis (OA) is the most common type of rheumatic disease and a leading cause of disability [9–11]. More than 10 percent of the world population have OA [12–15]. As OA progresses, severe joint pain limits patients' physical activity [16, 17]. Recently, OA has been recognized as a metabolic disease [18, 19] linked to metabolic syndrome [20–23]. Moreover, muscle weakness has been observed to be a frequent symptom among OA subjects compared to non-OA individuals [24, 25]. OA may contribute to the development of type 2 diabetes through complex processes involving metabolic syndrome, physical inactivity, and muscle weakness.

There exists evidence of an association between OA and diabetes or increased blood glucose [26–28]. However, most of the studies are cross-sectional and based on relatively small samples; thus, the causal association between OA and diabetes has not been established. In a prospective study, Nüesch et al. [29] showed that OA subjects are at increased risk for mortality due to diabetes, cardiovascular disease, and cancer. In a literature review, Hochberg reported higher mortality rates among OA cases compared with the general population [30].

In establishing causality, the use of incidence data and a cohort design are necessary to avoid the problem of reverse causality bias. To our knowledge, no prospective population-based study has been undertaken with a view to investigating the incidence of diabetes among persons with OA. We have conducted a prospective longitudinal study to examine the link between OA and diabetes using administrative health records, drawn from British Columbia (BC), Canada.

## 2. Materials and Methods

### 2.1. Administrative Data

Medical history of a large random sample (*n* = 577, 601) drawn from approximately 4 million residents registered in the Medical Service Plan (MSP) of BC, for the period from April 1991 to March 2009, was analyzed. MSP is a provincial health care plan in which approximately 99% of BC residents are registered. Both the BC Ministry of Health and the Population Data BC, who facilitate administrative data acquisition in BC, have approved access to and use of the data for this study [31]. This administrative database contains information on date of birth, sex, billing information for any health consultation, SES by area of residence, and hospital discharge records. To monitor deaths of individuals in the sample, the ministry linked vital statistics records to the database using personal health numbers. The database does not include records for patients treated at the emergency health care units.

### 2.2. Exposure and Outcome

The main exposure and outcome variables of interest were the administrative diagnoses of OA and diabetes, respectively. All OA cases were identified using the case definition that include at least two visits to a health professional within two years in different days or one discharge from the hospital with an International Classification of Disease 9th revision (ICD-9) code of 715 or International Statistical Classification of Diseases and Related Health Problems 10th revision (ICD-10) code of M15 to M19. These codes include symptomatic and radiographic OA in any joint except the spine. Using the same case definition as described for OA, diabetic cases were identified by looking at ICD-9 code of 250 or ICD-10 code of E11 to E14. A visit was defined as any service with the exclusion of diagnostic procedures and certain other procedures, such as dialysis/transfusion, anaesthesia, obstetrics, or therapeutic radiation. The second health professional visit or the hospital admission date was coded as the date of diagnosis. Individuals with a history of diabetes (any ICD code for diabetes mentioned above) before March 1996 (baseline) were excluded. After deleting prevalent diabetes cases, all OA cases aged 20 years and above, observed in the random sample during the period 1991–1996, were selected as the exposed group.

In the Canadian health care system, individuals with advanced stage of OA usually get referrals from their family physicians to orthopaedic surgeons (OS) for their surgical consultations. To examine the relationship between OA severity and the risk of diabetes, all exposed cases were divided into three groups according to disease severity: (1) OA diagnosis only, (2) at least one OS consultation, and (3) at least one total joint replacement (TJR) or revision before the baseline. OS consultations were identified by looking at the physician specialty code in the physician's billing data as well as in the hospital records. TJR cases were identified from hospital records using procedure codes (Canadian Classification of Diagnostic, Therapeutic and Surgical Procedures) for hip TJR and revisions 935.x, 936.5, 936.6, 936.7, and 936.8 and for knee TJR and revisions 934.0 and 934.1. Detailed explanation of these codes can be found elsewhere [32].

### 2.3. Nonexposed Group

For the purpose of selecting nonexposed group, one non-OA individual was matched randomly for each OA case, based on exact age, sex, and the year of OA diagnosis (index date), from the same random sample. Individuals in the non-OA comparison group never had a diagnosis for OA during the entire period (1991–2009), were aged 20 years and above, and were not diagnosed as diabetic prior to baseline.

### 2.4. Covariates and Confounders

The variables adjusted for in the analysis included age, sex, obesity, neighbourhood SES, and comorbid conditions related to diabetes. SES was assigned based on residential address linked to census data at the level of enumeration area (one or more adjacent blocks, up to 650 dwellings) and was grouped into 5 approximately equal income groups, from 1 (lowest) to 5 (highest), based on mean household income of those residents data [33]. Missing SES values (less than 5%) were imputed by generating random numbers from the binomial distribution. One of the most important risk factors for type 2 diabetes is obesity [4–6]. We identified overweight and obese individuals using the ICD-9 code of 278. In the entire random sample, we observed that 7.1% of the individuals aged 20 years or more were coded as obese during the period 1991–2009. Individuals' history of hypertension, hyperlipidemia, and chronic obstructive pulmonary disease (COPD) was assessed on or before the index date. These conditions were defined by visits to health care professionals or hospital admissions on or before baseline with the ICD-9 codes as follows: (1) hypertension, 401; (2) hyperlipidemia, 272; and (3) COPD, 490, 491, 494, and 496. In addition, Charlson comorbidity scores [34, 35] were calculated on or before baseline. The Charlson score was calculated by adding up the weights for each of the 19 comorbid conditions included in the algorithm.

### 2.5. BMI Imputation

Since body mass index (BMI) was not recorded in the administrative data, as part of sensitivity analysis, we imputed BMI categories using the Canadian Community Health Survey (CCHS) data cycles 1.1, 2.1, and 3.1. The CCHS is a large, cross-sectional, representative survey of the Canadian population, carried out by Statistics Canada. This survey contains nonoverlapping nationally representative data on health determinants, health status, and health services utilization. A detailed description of the survey design, sample, and interviewing procedures may be found elsewhere [36]. The method of imputation was designed to reproduce in our administrative dataset the associations of BMI with both OA and diabetes, observed in the CCHS. Individuals with OA were identified using self-reported OA and up to 3 non-OA individuals were randomly matched to each OA subject according to age, sex, and survey cycle. All OA and non-OA individuals were grouped according to the OA status, diabetes status, sex, and 10-year age groups. For each group, we calculated the proportion of individuals in each of the four BMI categories (kg/m^2^): underweight (<18.5), normal (18.5–24.9), overweight (25.0–29.9), and obese (≥30.0). Finally, we imputed the same proportion of individuals in each of the BMI categories from the CCHS data into our administrative data by matching on OA status, diabetes status, sex, and age.

### 2.6. Statistical Analysis

First, we performed a cross-sectional analysis with all individuals in the random sample. Using diabetes as a dependent variable, the unadjusted odds ratios (ORs) and the 95% confidence intervals (CIs) were calculated for OA and all other variables included in the analyses, by fitting logistic regression models. Next, in the prospective analysis, individuals were followed from the index date and continued until diabetes diagnosis, death, emigration, or the end of the study period (March 31, 2009), whichever came first. Cox proportional hazards (PH) models were fitted to estimate the relative risks (RRs) and the 95% CIs. The proportionality assumptions were assessed by checking the Kaplan-Meier curves as well as the proportionality tests *P* values from the multivariable PH models. The RRs were estimated by fitting Poisson regression models where the proportionality tests *P* values were found to be statistically significant. All analyses were performed using SAS V.9.3 (SAS Institute, Cary, NC, USA). The study was approved by the Behavioral Research Ethics Committee at the University of British Columbia, Vancouver, Canada.

## 3. Results

We observed 59,588 diabetic cases in the total random sample and the crude OR (95% CI) for OA was 2.93 (2.87–2.99) (data not shown). Between April 1991 and March 1996, we documented 19,089 OA cases. The mean age of OA cases was 61.6 years and 60.4% were women.  Table 1 shows the distribution of subjects according to exposure status (OA versus non-OA). At baseline, all variables except age and sex (matched variables) were significantly higher among the exposed group compared to the nonexposed group (*P* value < 0.05). During a 12-year mean follow-up period, corresponding to 465,399 person years, 5,231 new diabetes cases were observed. This gives an overall incidence rate (95% CI) of diabetes of 11.2 (10.90–11.50) per 1000 person years.

In the multivariable models, statistically significant interactions were observed between OA and age and OA and sex (*P* < 0.05). Therefore, separate analyses were performed using four strata: men aged 20–64 years, men aged ≥65 years, women aged 20–64 years, and women aged ≥65 years. We refer to these four groups as younger men, older men, younger women, and older women, respectively. During the follow-up, the incidence rate of diabetes was higher among men than women. Both adjusted and unadjusted RRs of diabetes according to the four subgroups are presented in Table 2. Since the proportionality test *P* values were significant for older women, RRs were estimated using the Poisson regression models.

OA showed a significantly increased risk of diabetes in all groups except older men in both crude and adjusted analyses. In the multivariable models, after adjusting for SES, obesity, history of COPD, hypertension, hyperlipidemia, and the Charlson score, the RRs (95% CI) were 1.16 (1.04–1.28), 1.27 (1.15–1.41), and 1.21 (1.08–1.35), respectively, for younger men, younger women, and older women. The RR was not significant for older men (RR = 0.94 (95% CI 0.82–1.09)). Lower SES, obesity, hypertension, and hyperlipidemia were associated with an increased risk of diabetes as expected. In the models, where obesity was replaced by the imputed BMI variable, similar RRs were observed for OA (data not shown).

**Figure 1:**
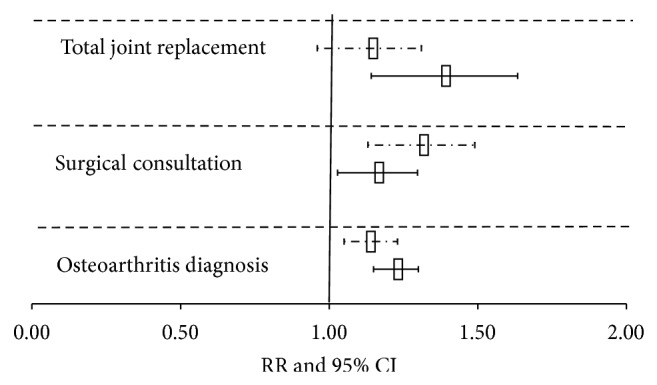
Adjusted relative risk (RR) and the 95% confidence interval (CI) of diabetes according to the severity of osteoarthritis. Box-plots with solid line represent cases of age 20–64 years and dash line represents cases of age 65 years and over. RRs were adjusted for age, sex, body mass index, socioeconomic status, chronic obstructive pulmonary disease, hypertension, hyperlipidemia, and Charlson index.

To check whether a dose-response relationship with disease severity exists among younger and older OA cases, we ran multivariable PH and Poisson regressions using OA diagnosis, surgical consultation, and TJR as exposure (Figure 1). Among OA cases, 1,811 (9.5%) had at least one TJR or revision and 3,080 (16%) had visits to orthopaedic surgeons before the baseline. Adjusted RR of diabetes was higher in the TJR group among younger OA cases (RRs (95% CI) were 1.22 (1.15–1.30), 1.15 (1.03–1.30), and 1.37 (1.14–1.63) for OA diagnosis, surgical consultation, and TJR groups, resp.). Among older OA cases (age 65 years and more) surgical consultation group showed the highest risk of diabetes (RRs (95% CI) were 1.13 (1.05–1.23), 1.30 (1.13–1.49), and 1.12 (0.96–1.31) for OA diagnosis, surgical consultation, and TJR groups, resp.).

## 4. Discussion

In this population-based prospective study with an average of 12 years of follow-up, we found that the risk of diabetes was higher in persons with OA compared to those without OA, after adjusting for confounding variables. The risk was significant in both younger and older women and in younger men, compared to their age-sex matched non-OA counterparts. We also observed that younger OA subjects who underwent TJR had a 37% higher risk of diabetes compared to non-OA individuals. Older men with OA showed no significant increased risk of diabetes.

There is some evidence that OA patients have a greater probability of developing diabetes. In a clinical and epidemiological survey, Cimmino and Cutolo [28] found that fasting glucose concentrations were significantly higher among OA cases compared to non-OA controls. Hart et al. [27] found an odds ratio of 1.95 of raised blood glucose among women with radiographic knee OA compared to non-OA women. In a cross-sectional study, Nieves-Plaza et al. [26] observed that adjusted odds ratio between OA and diabetes was 2.18 and the ratio was even higher among women. The statistically significant RRs observed in our prospective study may not be comparable with the odds ratios observed in earlier studies due to the difference in the study design.

Other published data have shown that OA patients are subject to increased mortality due to diabetes [29, 30]. Nüesch et al. [29] performed a population-based cohort study to examine cause and disease specific mortality in patients with OA of the hip and knee. They concluded that OA patients had a 95% higher rate of mortality due to diabetes compared to the general population. Deyo et al. [35] obtained a crude hazard ratio of 4.99 for mortality due to diabetes among OA patients. These studies had small sample sizes, and not all the relevant factors were adjusted for in the multivariable analyses. We analyzed a representative sample drawn from a prospectively collected administrative database in which total follow-up years covered the period from April 1991 to March 2009. By selecting OA cases during the years 1991 to 1996, we ensured that both the exposed and nonexposed groups had sufficient time to develop the outcomes of interest. In our prospective study, there was a minimum chance of reverse causality since we excluded prevalent diabetes cases at baseline. However, causality is very difficult to prove for cases having preclinical exposures and outcomes. The results were adjusted for age, sex, SES, obesity, and several conditions known to be associated with diabetes, such as COPD, hypertension, hyperlipidemia, and a number of severe conditions listed in the Charlson Comorbidity Index. Finally, we were able to perform separate analyses for specific age and sex categories.

The population-based design and the use of long-term medical records are the strengths of this study. An additional strength is that we used a large random sample of physician-diagnosed OA cases. Therefore, our results are generalizable to the Canadian population. Nonetheless, some limitations of the present study should be acknowledged. Both false negatives and false positives may occur in the diagnostic ICD-9 codes for OA and diabetes. However, these diagnostic criteria were validated previously for diabetes [37, 38] and for OA [39, 40]. Both Chen et al. [37] and Southern et al. [38] observed more than 70% sensitivity and positive predictive values for administrative diabetes codes. Lix et al. [39] obtained over 40% sensitivity and over 90% specificity of administrative OA diagnosis from Manitoba, Canada, compared to the self-reported CCHS data. Our case definition required two physician's visits over a two-year period or one hospital diagnosis and effectively reduced the number of false positives. To minimize false negatives, we selected nonexposed individuals who had not been diagnosed as OA during the entire period (1991–2009); we also eliminated diabetic cases before the baseline. Another limitation is the lack of information regarding physical activity and diet. However, diet has not been shown to be related to OA; therefore, it is not expected to be a confounder of this relationship. The percentage of obese subjects in our sample was slightly lower than the BC estimate [41]. Thus, for a sensitivity analysis, we imputed BMI from the CCHS data by matching randomly for age, sex, OA, and diabetes status. The RRs did not change when we replaced the obesity variable with the imputed BMI variable. We observed slightly higher RR of diabetes among young OA cases who underwent TJR, whereas RR among older OA cases who underwent TJR was not significant compared to non-OA subjects. A possible reason may be that although severe OA cases were advised for surgical treatments, a TJR might not prove that their OA cases were worse than someone who decided not to get a TJR.

While a high prevalence of OA among the elderly has long been recognized, little work has been done to find the association between OA and diabetes. Although the biological explanation for these associations has yet to be investigated, several possible causal paths can be hypothesized, including reduced mobility, metabolic syndrome, and muscle weakness. Obesity, hypertension, hyperlipidemia, and increased blood sugar are components of metabolic syndrome, all of which are common in both diabetic and OA patients. Our results were adjusted for some of these factors. In the present study, we were unable to adjust for possible confounding effects of physical activity and muscle weakness, and, therefore, further studies that include these variables are recommended. It should be noted, however, that these factors may not be confounders but rather intermediate variables in the causal path between OA and diabetes.

## 5. Conclusion

This large and prospective study has identified statistically significant associations between OA and diabetes. Our study suggests that younger men and women and older women with OA are at higher risks for developing diabetes compared to age-sex matched non-OA adults. However, these conclusions should be conditional on confirmation by future studies. More population-based studies are needed to understand the temporal ordering of the relationship between OA and the incidence of diabetes prospectively. These prospective longitudinal study results provide a rationale for undertaking further biological and epidemiological studies of increased blood sugar in persons with OA.

## Figures and Tables

**Table 1:** Distribution of variables by exposure (osteoarthritis) status and unadjusted relative risks (RRs) of diabetes observed in the prospective analysis.

Variable	No osteoarthritis (*n* = 19,089)	Osteoarthritis (*n* = 19,089)	Unadjusted RR (95% CI)
Diabetes	11.0	16.4	1.32 (1.25–1.39)

Sex (women)	60.4	60.4	1.01 (1.01-1.01)
Age (year)			
20–39	8.8	8.8	Reference
40–49	12.4	12.4	2.00 (1.72–2.32)
50–59	19.5	19.5	2.83 (2.47–3.25)
60–69	26.0	26.0	2.96 (2.59–3.39)
70–79	23.6	23.6	2.50 (2.17–2.87)
80+	9.8	9.8	1.76 (1.45–2.14)
Socioeconomic status			
1 (low)	23.1	23.5	1.40 (1.28–1.53)
2	21.5	21.8	1.36 (1.24–1.49)
3	18.4	18.9	1.28 (1.17–1.40)
4	18.3	18.4	1.13 (1.03–1.24)
5 (high)	18.8	17.4	Reference
Obesity	5.4	11.8	2.00 (1.86–2.16)
COPD	16.0	24.4	1.28 (1.20–1.37)
Hypertension	31.5	40.2	1.92 (1.82–2.03)
Hyperlipidemia	9.3	11.4	1.44 (1.33–1.55)
Charlson score mean (SD)	0.94 (1.83)	1.07 (1.58)	1.06 (1.04–1.08)

The numbers are percent unless otherwise mentioned. OA: osteoarthritis; COPD: chronic obstructive pulmonary disease.

**Table 2:** Age-sex specific incidence rate per 1000 person years, relative risks (RRs), and the 95% confidence intervals (CIs) of diabetes mellitus (DM).

Variables	Men		Women	
	Age < 65 years (*n* = 9,086)	Age ≥ 65 years (*n* = 6,040)	Age < 65 years (*n* = 11,184)	Age ≥ 65 years (*n* = 11,868)
DM incidence rate (1,000)	12.5 (11.9–13.1)	13.0 (12.1–13.9)	10.2 (9.7–10.8)	10.4 (9.9–11.0)

Exposure	RR (95% CI)	RR (95% CI)	RR (95% CI)	RR (95% CI)
OA unadjusted	1.32 (1.19–1.46)	0.99 (0.86–1.14)	1.53 (1.38–1.70)	1.32 (1.19–1.48)
OA adjusted	1.16 (1.04–1.28)	0.94 (0.82–1.09)	1.27 (1.15–1.41)	1.21 (1.08–1.35)
SES				
1 (low)	1.40 (1.19–1.66)	1.18 (0.95–1.46)	1.57 (1.33–1.85)	1.16 (0.98–1.39)
2	1.27 (1.08–1.50)	1.18 (0.94–1.47)	1.53 (1.30–1.80)	1.17 (0.98–1.40)
3	1.42 (1.20–1.67)	0.91 (0.72–1.16)	1.35 (1.14–1.61)	1.18 (0.98–1.43)
4	1.07 (0.90–1.27)	1.09 (0.86–1.37)	1.12 (0.94–1.34)	1.15 (0.95–1.39)
5 (high)	Reference	Reference	Reference	Reference
Obesity	1.95 (1.70–2.24)	1.62 (1.25–2.11)	1.73 (1.54–1.95)	1.98 (1.69–2.32)
COPD	1.22 (1.06–1.41)	1.01 (0.84–1.20)	1.20 (1.05–1.36)	1.13 (0.99–1.29)
Hypertension	2.14 (1.92–2.38)	1.43 (1.24–1.65)	2.20 (1.99–2.44)	1.45 (1.30–1.63)
Hyperlipidemia	1.33 (1.16–1.53)	1.31 (1.07–1.60)	1.18 (1.02–1.36)	1.33 (1.14–1.55)
Charlson score	1.01 (0.96–1.06)	0.99 (0.95–1.03)	1.02 (0.97–1.07)	0.96 (0.93–1.00)

COPD: chronic obstructive pulmonary disease; SES: socioeconomic status; OA: osteoarthritis.
